# Preoperative Inflammatory Blood Biomarkers in Sinonasal Carcinoma: A Scoping Review

**DOI:** 10.3390/medicina62061046

**Published:** 2026-05-28

**Authors:** Andrea Migliorelli, Marianna Manuelli, Andrea Ciorba, Francesco Stomeo, Stefano Pelucchi, Chiara Bianchini

**Affiliations:** ENT & Audiology Unit, Department of Neurosciences, University Hospital of Ferrara, 44100 Ferrara, Italy

**Keywords:** sinonasal carcinoma, sinonasal squamous cell carcinoma, inflammatory biomarkers, neutrophil-to-lymphocyte ratio (NLR), PLR, ALI, SII

## Abstract

*Background and Objectives*: Sinonasal carcinomas are heterogeneous malignancies often associated with poor prognosis because of the locally advanced stage at presentation and high recurrence rates. Easily accessible prognostic biomarkers are therefore of increasing clinical interest. Systemic inflammatory blood markers have shown prognostic value in several solid tumors, but their role in sinonasal carcinomas remains unclear. *Materials and Methods*: A scoping review was performed according to PRISMA-ScR recommendations. The PubMed/MEDLINE, Scopus, and Embase databases were searched from inception to March 2026. Original English-language studies evaluating preoperative or pre-treatment inflammatory blood biomarkers in sinonasal malignant tumors were included. *Results*: Six retrospective single-center studies met the inclusion criteria, comprising 1049 patients. All studies evaluated biomarkers before treatment initiation in cohorts managed primarily with upfront surgery. The neutrophil-to-lymphocyte ratio (NLR) was the most frequently investigated biomarker and the one most commonly associated with adverse oncologic outcomes, including poorer survival, advanced stage, and increased recurrence risk. Additional biomarkers included the platelet-to-lymphocyte ratio, advanced lung cancer inflammation index (ALI), systemic immune-inflammation index (SII), and other composite scores. Preliminary evidence suggests that ALI and SII may provide additional prognostic information beyond isolated inflammatory ratios, although current evidence remains limited. *Conclusions*: Preoperative inflammatory blood biomarkers, particularly NLR, appear to be promising, low-cost, and widely accessible prognostic tools for risk stratification in sinonasal carcinomas. However, current evidence is limited by retrospective study design, heterogeneous cohorts, and non-standardized cut-off values. Prospective multicenter studies are needed before routine clinical implementation can be recommended.

## 1. Introduction

Sinonasal carcinomas represent a rare and heterogeneous group of malignant neoplasms arising from the nasal cavity and paranasal sinuses, accounting for less than 5% of all head and neck malignancies [1]. Although uncommon, these tumors remain clinically relevant because of their frequently aggressive biological behavior, anatomical proximity to critical structures, and persistent therapeutic challenges [2,3]. The most common histological subtype is sinonasal squamous cell carcinoma (SNSCC), followed by a wide spectrum of rarer entities, including adenocarcinomas, neuroendocrine carcinomas, sinonasal undifferentiated carcinoma, olfactory neuroblastoma, mucosal melanoma, and salivary-type malignancies [2,4,5].

One of the major clinical issues associated with sinonasal carcinomas is delayed diagnosis. Early symptoms such as unilateral nasal obstruction, epistaxis, rhinorrhea, headache, facial pain, or reduced olfaction are often nonspecific and may mimic benign inflammatory sinonasal conditions leading to an underestimation of the condition [6,7]. As a consequence, many patients are diagnosed only after progression to locally advanced disease, frequently with extension to the orbit, skull base, cranial nerves, dura mater, or adjacent neurovascular structures. Such presentations often require highly complex management and are associated with worse oncologic outcomes [6,7].

At present, surgery remains the cornerstone of treatment in most resectable cases and may be performed through endoscopic, open craniofacial, or combined approaches depending on tumor extent and anatomical involvement [8]. Surgical treatment is commonly integrated with postoperative radiotherapy and, in selected advanced-stage tumors or aggressive histological subtypes, chemotherapy or multimodal protocols [8]. Despite improvements in skull base surgery techniques, image guidance, reconstructive techniques, intensity-modulated radiotherapy, proton therapy, and systemic treatments, prognosis remains suboptimal. Five-year survival rates are highly variable according to stage and histology but are frequently reported below 50%, while local recurrence continues to represent the leading cause of treatment failure [2,3,9,10].

Considering this scenario, improving prognostic stratification at the time of diagnosis is of major importance. Current treatment planning mainly relies on TNM staging, histopathological subtype, tumor grade, surgical margins, perineural invasion, lymphovascular invasion, and radiological extension [11]. However, patients with apparently similar clinicopathological features may experience markedly different clinical courses, suggesting that conventional prognostic variables alone may not fully capture the biological heterogeneity of these tumors [5,12]. Consequently, there is growing interest in additional biomarkers able to improve risk assessment and personalize treatment strategies.

Emerging technologies such as radiomics and artificial intelligence are also being investigated in sinonasal malignancies for diagnostic and prognostic purposes [13,14]. However, despite promising preliminary findings, these approaches still require broader validation and currently remain mainly investigational.

Besides these advanced approaches, increasing attention has also focused on more readily available and cost-effective biomarkers that can be incorporated into routine clinical workflows. In this context, systemic inflammation has emerged as an important contributor to cancer progression. It is now widely recognized that tumor cells interact dynamically with the host immune–inflammatory system, promoting angiogenesis, immune escape, tumor growth, local invasion, and metastatic dissemination. The balance between pro-tumoral inflammatory pathways and anti-tumoral immune surveillance may therefore be reflected by simple hematological parameters derived from peripheral blood counts [15,16,17].

Several inflammatory blood biomarkers have shown prognostic significance across multiple solid tumors [18,19,20,21]. Among the most commonly studied are the neutrophil-to-lymphocyte ratio (NLR), platelet-to-lymphocyte ratio (PLR), lymphocyte-to-monocyte ratio (LMR), systemic immune-inflammation index (SII), prognostic nutritional index (PNI), and advanced lung cancer inflammation index (ALI) [15]. These markers are inexpensive, reproducible, and widely available, making them particularly attractive for daily clinical practice. In head and neck squamous cell carcinoma, elevated NLR and related indices have repeatedly been associated with poorer survival, higher recurrence rates, treatment resistance, and reduced response to therapy [15,16,17].

However, evidence derived from broader head and neck squamous cell carcinoma cohorts may not be directly applicable to sinonasal malignancies because of their marked histological heterogeneity, unique anatomical environment, and distinct multidisciplinary treatment strategies.

From a practical standpoint, inflammatory blood biomarkers may be especially relevant in sinonasal carcinomas. These tumors often require complex multidisciplinary decision-making, and pre-treatment risk assessment may influence surgical planning, need for adjuvant treatment, intensity of postoperative surveillance, and patient counseling. Moreover, because routine blood tests are already part of standard preoperative work-up, these biomarkers could be implemented with minimal additional costs.

Nevertheless, despite increasing evidence in other oncologic settings, the specific role of inflammatory blood biomarkers in sinonasal carcinomas remains less clearly defined. Published studies are relatively few, often retrospective, and include heterogeneous patient populations and tumor histologies. Furthermore, the rarity of sinonasal cancers makes prospective validation particularly challenging.

The aim of the present scoping review was to critically analyze the currently available evidence regarding the prognostic value of pre-treatment inflammatory blood biomarkers in sinonasal carcinomas. Particular attention was given to their potential use in preoperative risk stratification, prediction of oncologic outcomes, and future integration into personalized management strategies.

## 2. Materials and Methods

**Figure 1:**
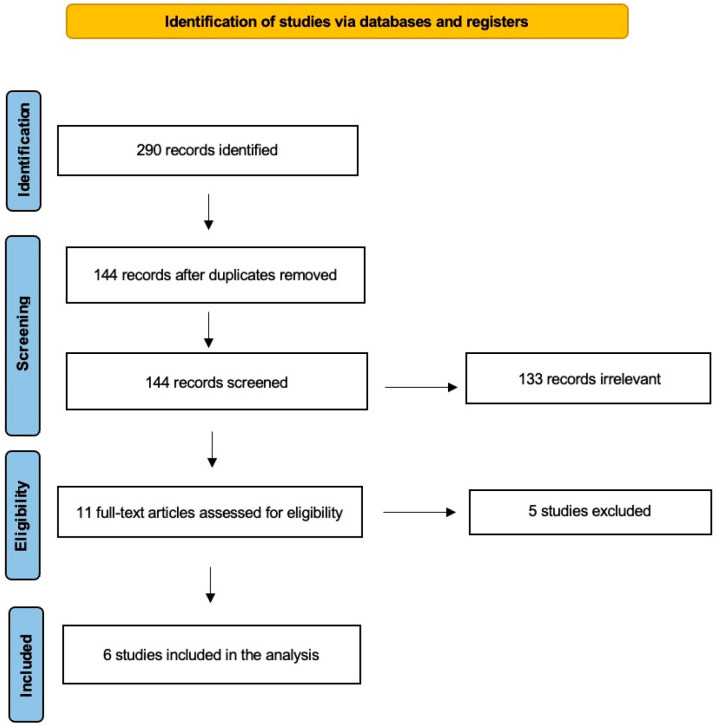
The literature review performed using PRISMA guidelines for scoping review.

A systematic literature search of English-language studies investigating the role of inflammatory blood biomarkers in sinonasal carcinomas was performed using the PubMed/MEDLINE, Scopus, and Embase databases. The search was conducted from database inception to March 2026. The review process followed the Preferred Reporting Items for Systematic Reviews and Meta-Analyses extension for Scoping Reviews (PRISMA-ScR) [22] (Figure 1). A scoping review methodology was selected because currently available evidence on inflammatory blood biomarkers in sinonasal malignancies remains limited and heterogeneous regarding histological subtypes, biomarkers investigated, cut-off values, and reported oncologic outcomes.

The search strategy combined terms related to sinonasal malignancies with terms associated with systemic inflammatory biomarkers. Specifically, keywords included sinonasal, nasal cavity, paranasal sinus, paranasal sinuses, Paranasal Sinus Neoplasms, and Nasal Cavity Neoplasms, combined with neutrophil-to-lymphocyte ratio, NLR, platelet-to-lymphocyte ratio, PLR, lymphocyte-to-monocyte ratio, LMR, monocyte-to-lymphocyte ratio, MLR, systemic immune-inflammation index, SII, systemic inflammation response index, and SIRI. Search syntax was adapted according to the requirements of each database.

Studies were considered eligible if they met the following inclusion criteria: (i) original full-text studies published in English; (ii) studies including patients affected by malignant sinonasal tumors; (iii) studies evaluating preoperative peripheral blood inflammatory or immune–inflammatory biomarkers; and (iv) studies investigating the prognostic or clinical significance of such biomarkers. Exclusion criteria were: (i) case reports, conference abstracts, letters, editorials, and narrative reviews; (ii) studies including only benign sinonasal lesions; (iii) studies lacking specific data on inflammatory biomarkers; (iv) studies in which biomarkers were not assessed before treatment initiation; and (v) duplicated or overlapping patient cohorts.

Studies exclusively including recurrent tumors or pediatric populations were not identified among the eligible studies. Non-surgical cohorts were not formally excluded; however, all included studies primarily involved patients undergoing upfront surgical treatment.

The initial database search identified 290 records. After duplicate removal, 144 articles remained for title and abstract screening. Following preliminary evaluation, 11 studies underwent full-text assessment for eligibility. At the end of the selection process, six studies fulfilled all inclusion criteria and were included in the present scoping review [5,12,23,24,25,26].

Two reviewers (AM and MM) independently screened titles, abstracts, and full texts of potentially eligible studies. Disagreements regarding study inclusion were resolved through discussion with a senior reviewer (CB) until consensus was achieved.

Data extracted from each study included author, year of publication, study design, sample size, tumor histology, inflammatory biomarkers investigated, biomarker cut-off values, timing of blood collection, exclusion criteria, treatment modality, use of neoadjuvant therapy when reported, and oncologic outcomes.

Special consideration was given to the timing of biomarker assessment relative to treatment initiation and to the primary treatment modality.

Quantitative meta-analysis and formal risk-of-bias assessment were not performed because the primary aim of this scoping review was to map the available evidence and identify current research gaps rather than to conduct quantitative synthesis, particularly in light of the substantial heterogeneity among included studies in terms of histological subtypes, inflammatory biomarkers evaluated, cut-off values, treatment strategies, outcome measures, and statistical approaches.

Therefore, findings were synthesized descriptively and narratively.

The protocol for this review was registered on the Open Science Framework (OSF) Registries with the Registration https://osf.io/w84xs (accessed on 23 April 2026).

## 3. Results

### 3.1. Study Selection and General Characteristics

A total of six full-text studies met the predefined inclusion criteria and were included in the present scoping review [5,12,23,24,25,26]. Overall, the selected studies comprised 1049 patients affected by sinonasal malignancies, with individual sample sizes ranging from 41 to 473 cases. All included investigations had a retrospective observational design and were conducted in single tertiary referral centers.

The main characteristics of the included studies and their principal findings are summarized in Table 1 and Table 2.

Three studies specifically focused on sinonasal squamous cell carcinoma [23,24,25,26], which was therefore the most frequently investigated histological subtype. Two additional studies analyzed more heterogeneous cohorts including multiple sinonasal malignancies and skull base tumors with different histologies [5,12]. One study was exclusively dedicated to olfactory neuroblastoma [25].

Surgery represented the primary treatment modality in all included studies. Patients were managed with upfront resection, either alone or followed by postoperative radiotherapy and/or chemotherapy according to pathological stage, histology, margin status, or multidisciplinary indications. Biomarkers were calculated using peripheral blood samples obtained during the routine preoperative work-up. Most included studies evaluated biomarkers before treatment initiation and excluded patients who had received neoadjuvant chemotherapy or radiotherapy [5,23,26].

### 3.2. Biomarkers Evaluated

The NLR was assessed in all six studies and was therefore the most extensively investigated inflammatory marker.

Other biomarkers included PLR, LMR, ALI, SII, albumin-to-globulin ratio (AGR), prognostic nutritional index (PNI) and serum albumin.

Considerable variability was observed regarding threshold values used to define abnormal biomarker levels. Reported NLR cut-offs ranged from 2.6 in the study by Turri-Zanoni et al. to values close to 7.0 in the study by Valero et al., where the upper fifth percentile was used to define NLR-high patients [5,12]. Zhong et al. used the median value of their cohort as the threshold [23]. PLR cut-off was reported as 156.9 in one study [5]. For ALI and SII, proposed thresholds were 27.80–29.5 and 791.35, respectively [24,26].

### 3.3. Neutrophil-to-Lymphocyte Ratio

NLR was the biomarker most frequently associated with adverse oncologic outcomes across the included retrospective studies.

In the retrospective study by Zhong et al., 147 patients with surgically treated sinonasal squamous cell carcinoma were evaluated [23]. Multivariate Cox regression analysis demonstrated that pathological T stage, pathological N stage, and preoperative NLR were independent predictors of overall survival. The same variables also independently predicted disease-free survival and disease-specific survival, suggesting a stable prognostic effect of elevated NLR across multiple clinically relevant endpoints.

Turri-Zanoni et al. [5] analyzed 215 patients with primary sinonasal malignancies treated endoscopically and stratified them into five histological groups: adenocarcinoma, carcinoma (including squamous cell carcinoma and adenoid cystic carcinoma), olfactory neuroblastoma, mucosal melanoma, and miscellaneous tumors. Among patients with epithelial tumors (adenocarcinoma and carcinoma groups), elevated pre-treatment NLR and PLR values were significantly associated with poorer overall survival and disease-free survival. In contrast, no statistically significant differences in survival outcomes according to NLR or PLR levels were observed in patients with olfactory neuroblastoma, mucosal melanoma, or miscellaneous tumors. When stratified by tumor stage, no significant associations were found in early-stage disease (pT1–pT2), whereas patients with advanced tumors (pT3–pT4) and higher NLR or PLR values showed significantly shorter overall survival and disease-free survival. In multivariate Cox regression analysis adjusted for age, clinical stage, and histological subtype, both NLR and PLR emerged as independent prognostic factors for disease-free survival, but not for overall survival. Specifically, patients with NLR < 2.6 and PLR < 156.9 showed a significantly lower risk of recurrence.

Valero et al. [12] reported the largest cohort included in the present review, comprising 473 patients with sinonasal and skull base malignancies treated surgically. During follow-up, 104 patients developed distant recurrence. Patients with metastatic failure showed a significantly higher prevalence of elevated NLR compared with those without distant relapse. In multivariable analysis, NLR remained an independent predictor of distant recurrence together with melanoma histology, advanced T classification, and nodal disease.

In the study by Brkic et al. [24], which included 41 patients with SNSCC, high pretherapeutic NLR values were significantly associated with worse overall survival. However, no independent association with disease-free survival was demonstrated after multivariate adjustment.

Mocharnuk et al. [25] investigated 44 patients with olfactory neuroblastoma treated at initial disease presentation. Patients with an advanced Kadish stage (C/D) showed significantly higher NLR values compared with those presenting with lower-stage disease (A/B). Nevertheless, no significant relationship was found between NLR and Hyams grade, recurrence, or mortality.

Overall, these findings suggest that NLR may reflect disease aggressiveness and unfavorable oncologic behavior across different sinonasal tumor subtypes, although stronger evidence is currently available for squamous cell carcinoma.

### 3.4. Platelet-to-Lymphocyte Ratio

PLR was evaluated less frequently than NLR, but available evidence suggested a potentially relevant prognostic role.

Turri-Zanoni et al. [5] demonstrated that elevated pretreatment PLR values were associated with shorter overall survival and disease-free survival, particularly in epithelial and advanced-stage sinonasal cancers. In the same study, PLR values below 156.9 were associated with a significantly lower risk of recurrence, similarly to what was observed for NLR.

PLR was also included in the broader comparative biomarker analysis performed by Wu et al. [26] in patients with SNSCC. However, in that study, ALI and SII demonstrated superior discriminatory and prognostic performance.

Although data remain limited, PLR may represent an additional low-cost marker worthy of further validation.

### 3.5. Composite Inflammatory and Nutritional Indices

More recent studies investigated composite indices integrating systemic inflammation and nutritional status.

Brkic et al. [24] evaluated pretherapeutic NLR, serum albumin, body mass index (BMI), and ALI in a cohort of 41 patients with sinonasal squamous cell carcinoma. Low ALI values were associated with worse overall survival, whereas BMI emerged as the strongest prognostic factor. Patients with lower BMI experienced significantly poorer overall survival and disease-free survival, and BMI remained independently associated with overall survival in multivariate analysis. None of the investigated biomarkers retained independent significance for disease-free survival.

Wu et al. [26] analyzed 129 patients with SNSCC and compared several indices, including NLR, PLR, LMR, AGR, ALI, SII, and PNI. Among these variables, ALI and SII demonstrated the highest prognostic performance. Based on these findings, the authors developed a prognostic nomogram incorporating tumor stage, ALI, and primary tumor site. Internal validation using calibration plots and decision curve analysis suggested satisfactory predictive accuracy and potential clinical utility.

These findings indicate that multidimensional biomarkers integrating inflammatory and nutritional parameters may outperform isolated leukocyte-derived ratios.

### 3.6. Heterogeneity Across Studies

Substantial heterogeneity was observed among the included studies.

Differences were present in patient populations, histological subtypes, treatment strategies, biomarkers investigated, cut-off values, statistical approaches, and outcome measures. Some studies focused exclusively on sinonasal squamous cell carcinoma, whereas others included mixed sinonasal malignancies, skull base cancers, or olfactory neuroblastoma.

Likewise, oncologic endpoints varied considerably and included overall survival, disease-free survival, disease-specific survival, distant recurrence, recurrence risk, and correlations with staging or grading systems.

Earlier studies mainly focused on NLR and PLR, whereas more recent investigations increasingly explored composite indices such as ALI and SII.

Because of these methodological and clinical differences, direct quantitative comparison among studies was not feasible, and pooled statistical analysis was considered inappropriate.

## 4. Discussion

The present scoping review critically summarized the currently available evidence regarding the prognostic role of inflammatory blood biomarkers in sinonasal carcinomas. Although the number of eligible studies remains limited, several relevant observations emerge from the published literature [5,12,23,24,25,26].

Before discussing the available clinical evidence, it is important to consider the biological rationale supporting the prognostic role of systemic inflammatory biomarkers in cancer. The rationale supporting the use of inflammatory biomarkers in cancer prognosis is biologically plausible and increasingly supported by translational research [27,28]. Cancer progression is not determined solely by intrinsic tumor genetics, but also by dynamic interactions between malignant cells and the host immune–inflammatory environment [29,30,31]. Neutrophils may promote tumor growth through secretion of cytokines, proteases, angiogenic mediators, and immunosuppressive factors [32,33,34]. Conversely, lymphocytes play a key role in antitumor immune surveillance and cytotoxic response [35]. Therefore, a relative increase in neutrophils combined with lymphopenia may reflect a biological environment favorable to tumor progression. This concept underlies the clinical interest in NLR as a surrogate marker of host–tumor interaction [15,17].

Interest in systemic inflammatory markers has progressively expanded even in this rare and heterogeneous oncologic setting. In addition to NLR, more limited and preliminary evidence suggests that composite indices integrating inflammation and nutritional status, such as ALI and SII, may provide additional prognostic information beyond simple leukocyte-derived ratios.

A particularly noteworthy aspect of the current evidence is the relative homogeneity of the clinical setting analyzed [5,12,23,24,25,26]. All studies included evaluated biomarkers before treatment initiation and focused on cohorts managed primarily with upfront surgery, with adjuvant radiotherapy or chemoradiotherapy administered when indicated. This feature enhances the practical relevance of the available findings.

Indeed, if prospectively validated, biomarkers measured during the routine preoperative work-up may eventually contribute to surgical decision-making, patient counseling, postoperative surveillance planning, and multidisciplinary treatment discussions.

In the present review, NLR was evaluated across all included studies and emerged as the inflammatory biomarker most frequently associated with adverse oncologic outcomes [5,12,23,24,25,26]. Elevated preoperative NLR was generally associated with poorer survival, advanced disease stage, and increased recurrence risk, particularly in sinonasal squamous cell carcinoma and other epithelial malignancies. Several studies also reported that these associations remained significant after adjustment for tumor stage and other clinicopathological variables in multivariate analyses [5,12,23]. In contrast, prognostic significance appeared less consistent in non-epithelial tumors such as olfactory neuroblastoma and mucosal melanoma. Overall, the most clinically interpretable evidence currently concerns surgically treated sinonasal squamous cell carcinoma.

These observations are also coherent with the wider literature on head and neck squamous cell carcinoma and other rare skull base malignancies. Several multicenter studies have shown that elevated pre-treatment NLR is associated with reduced survival, increased recurrence risk, poorer treatment response, and more aggressive pathological features in oral cavity, laryngeal, hypopharyngeal, and oropharyngeal cancers [15,16,19]. In surgically treated HPV-negative head and neck squamous cell carcinoma, persistently elevated postoperative inflammatory markers have also been associated with unfavorable prognosis [17]. The biological consistency between sinonasal tumors and other head and neck malignancies therefore reinforces the likelihood that systemic inflammatory response carries genuine prognostic significance in sinonasal cancers as well.

Another relevant trend emerging from the reviewed studies is the progressive transition from simple biomarkers toward multidimensional composite indices [24,26]. Although NLR remains attractive because of its simplicity and immediate availability, cancer prognosis is influenced by several interacting variables, including nutritional status, systemic catabolism, albumin levels, body composition, and immune function. Composite scores may therefore provide a more complete estimate of host frailty and oncologic resilience.

Among these indices, ALI deserves particular attention. This score combines body mass index, serum albumin, and NLR, thereby integrating inflammatory burden with nutritional reserve. In the studies included in this review, low ALI values were associated with poorer outcomes, while in the most recent comparative analysis ALI showed superior prognostic performance compared with several traditional inflammatory ratios [24,26]. Similar findings have been reported in lung cancer, gastrointestinal malignancies, and head and neck squamous cell carcinoma, where ALI has emerged as a robust predictor of survival and treatment tolerance [16,36,37].

Likewise, SII, calculated using platelet, neutrophil, and lymphocyte counts, demonstrated promising prognostic value in the most recent sinonasal carcinoma cohort [26]. Since platelets may contribute to tumor cell survival, endothelial adhesion, angiogenesis, and metastatic dissemination, combining platelet burden with neutrophil and lymphocyte counts may better capture the complexity of cancer-related inflammation [38,39,40,41]. Although evidence in sinonasal tumors remains preliminary, these observations are encouraging.

From a practical standpoint, inflammatory blood biomarkers possess several advantages that make them attractive for clinical use. They are inexpensive, widely available, rapidly obtainable, and reproducible across institutions. No additional invasive procedures are required, since these parameters are derived from routine preoperative blood tests already included in standard patient work-up. This aspect is particularly relevant in rare tumors such as sinonasal carcinomas, where sophisticated molecular testing may not always be readily accessible.

If prospectively validated, these biomarkers could support several clinically meaningful applications. Patients with unfavorable inflammatory profiles might benefit from intensified postoperative surveillance, closer radiological follow-up, more careful selection for adjuvant treatment, or enrollment in clinical trials. Conversely, patients with low-risk biomarker profiles might potentially avoid unnecessary overtreatment. Such an approach would align with the broader movement toward precision oncology and personalized treatment pathways.

Nevertheless, despite these promising aspects, the currently available evidence should be interpreted cautiously. First, the number of published studies remains small, with only six eligible investigations identified in the present review. This limitation is not unexpected given the rarity of sinonasal carcinomas, but it inevitably restricts the strength of available conclusions. Second, all included studies had retrospective single-center designs. Such methodology is intrinsically vulnerable to selection bias, missing data, treatment heterogeneity, immortal time bias, variability in laboratory measurements, and confounding variables that may not be fully controlled in multivariate models.

In addition, inflammatory biomarkers may be influenced by several non-oncologic factors, including infections, corticosteroid use, smoking status, autoimmune diseases, nutritional status, and other systemic inflammatory conditions, which were not consistently reported across the included studies. Furthermore, although all included studies evaluated biomarkers before treatment initiation, the exact timing of blood collection relative to surgery or other therapeutic interventions was not uniformly standardized or consistently reported. Third, considerable heterogeneity was observed regarding patient populations, histological subtypes, treatment strategies, biomarker thresholds, and outcome measures. Some studies focused exclusively on sinonasal squamous cell carcinoma, whereas others included mixed sinonasal cancers, skull base tumors, or olfactory neuroblastoma. This diversity complicates direct comparison and limits generalizability. Fourth, no universally accepted cut-off values currently exist for the most studied biomarkers, particularly NLR. Reported thresholds varied substantially across studies, often derived from cohort medians or internal ROC analyses. This variability reduces immediate clinical applicability and makes external validation essential before routine adoption. Finally, publication bias and small-study effects cannot be excluded, as studies reporting significant prognostic associations are generally more likely to be published than negative or inconclusive investigations.

Considering these limitations, future research should focus on multicenter prospective validation studies involving larger and histologically stratified patient cohorts. Shared methodological criteria and standardized threshold values are urgently needed. Moreover, integrated prognostic models combining blood biomarkers with clinical stage, pathological factors, molecular alterations, and radiological features should be developed and externally validated. Future studies should also determine whether inflammatory biomarkers provide incremental prognostic value beyond established staging systems and clinicopathological risk factors.

Another promising area of investigation concerns dynamic biomarker assessment. While the studies included in this review focused almost exclusively on pre-treatment values, changes occurring after surgery, during radiotherapy, or throughout follow-up may provide additional prognostic information. Serial monitoring of inflammatory markers could potentially identify minimal residual disease, early relapse, treatment-related systemic stress, or evolving resistance patterns.

Overall, the currently available evidence suggests that inflammatory blood biomarkers—particularly NLR—may represent promising prognostic tools in surgically treated sinonasal carcinomas. More recent multidimensional indices such as ALI and SII may further improve risk stratification. However, before these markers can be incorporated into routine clinical practice, robust prospective external validation remains necessary.

## 5. Conclusions

The present scoping review highlights that inflammatory blood biomarkers may represent promising prognostic tools in sinonasal carcinomas, particularly in patients undergoing surgery as the primary treatment. Among the currently available markers, NLR appears to be the most extensively investigated biomarker and the one most frequently associated with adverse oncologic outcomes across the available retrospective studies, including survival, recurrence risk, distant failure, and disease aggressiveness. At present, the available evidence should be considered primarily hypothesis-generating rather than practice-changing.

More recent composite indices, such as ALI and SII, may provide additional prognostic information by integrating systemic inflammation, nutritional status, and host biological reserve. These multidimensional markers could better reflect the complex interaction between tumor behavior and patient-related factors than isolated leukocyte-derived ratios alone.

However, the currently available evidence is based exclusively on retrospective, predominantly single-center studies with relatively small and heterogeneous cohorts, while the absence of standardized cut-off values currently limits clinical applicability. Therefore, routine implementation of these biomarkers in sinonasal carcinoma management cannot yet be recommended. Prospective multicenter studies are needed to validate current findings, establish reproducible thresholds, and develop integrated prognostic models combining inflammatory biomarkers with clinicopathological and molecular parameters.

Nevertheless, because of their simplicity, low cost, and wide availability, inflammatory blood biomarkers remain an area of considerable interest and may contribute in the future to more personalized treatment planning, tailored surveillance strategies, and improved risk stratification in patients with sinonasal carcinomas.

## Figures and Tables

**Table 1:** Main characteristics of the studies included in the scoping review.

Author, (Year)	Country	Histology	No. of Patients	Inflammatory Markers Evaluated	Cut-Off Values	Outcomes Assessed
Turri-Zanoni et al. (2016) [5]	Italy	SC (mixed histologies)	215	NLR, PLR	NLR: 2.6 PLR: 156.9	OS, DFS
Zhong et al. (2019) [23]	China	SNSCC	147	NLR	NLR: 4.25 *	OS, DFS, DSS
Brkic et al. (2019) [24]	Austria	SNSCC	41	NLR, albumin, ALI	NLR: 3.5 * ALI: 29.5 *	OS, DFS
Valero et al. (2021) [12]	USA	SC (mixed histologies)	473	NLR	NLR: 7 **	DR, OS, DSS
Mocharnuk et al. (2024) [25]	USA	ONB	44	NLR	Continuous variable	Kadish stage, Hyams grade, DFS, OS
Wu et al. (2025) [26]	China	SNSCC	129	NLR, PLR, LMR, AGR, ALI, SII, PNI	ALI: 27.80 SII: 791.35	OS, prognostic nomogram

Abbreviation: SC: sinonasal cancer; SNSCC: Sinonasal squamous cell carcinoma, ONB: Olfactory neuroblastoma, NLR: neutrophil-to-lymphocyte ratio, PLR: platelet-to-lymphocyte ratio, LMR: lymphocyte-to-monocyte ratio, AGR: albumin-to-globulin ratio, ALI: advanced lung cancer inflammation index, SII: systemic immune-inflammation index, PNI: prognostic nutritional index, OS: Overall survival, DFS: disease-free survival, DSS: disease-specific survival, DR: Distant recurrence, *: median value, **: Top 5 percentile.

**Table 2:** Main findings and limitations of the included studies.

Authors, Year	Main Findings	Clinical Relevance	Main Limitations
Turri-Zanoni et al. (2016) [5]	High pretreatment NLR and PLR were associated with worse OS and DFS, especially in epithelial advanced-stage tumors. NLR < 2.6 and PLR < 156.9 correlated with lower recurrence risk.	Suggests simple blood-based prognostic stratification in surgically treated sinonasal cancers.	Retrospective design; single-center study.
Zhong et al. (2019) [23]	Preoperative NLR was an independent prognostic factor for OS, DFS, and DSS in SNSCC.	Supports NLR as robust biomarker in SNSCC	Retrospective design; median-based cut-off not externally validated; single-center study.
Brkic et al. (2019) [24]	High NLR and low ALI were associated with worse OS; BMI was the strongest independent prognostic factor. No marker independently predicted DFS.	Highlights the interaction between inflammation and nutritional status.	Very small sample size; limited statistical power; single-center retrospective cohort.
Valero et al. (2021) [12]	High NLR independently predicted distant recurrence. Melanoma histology, advanced T stage, and nodal disease were additional risk factors.	Suggests NLR may reflect host immune status and metastatic risk.	Highly heterogeneous cohort; retrospective design; single-center study
Mocharnuk et al. (2024) [25]	Elevated NLR was associated with advanced Kadish stage, but not with recurrence or mortality.	Indicates a possible role of NLR in staging rather than survival prediction in ONB.	Small cohort; retrospective study; single-center study
Wu et al. (2025) [26]	ALI and SII showed the best prognostic performance. Nomogram including tumor stage, ALI, and primary site demonstrated good calibration.	Composite indices may outperform isolated biomarkers such as NLR.	No external validation cohort; retrospective design; single-center population.

Abbreviation: SNSCC: Sinonasal squamous cell carcinoma, ONB: Olfactory neuroblastoma, NLR: neutrophil-to-lymphocyte ratio, PLR: platelet-to-lymphocyte ratio, BMI: body mass index, ALI: advanced lung cancer inflammation index, SII: systemic immune-inflammation index, OS: Overall survival, DFS: disease-free survival, DSS: disease-specific survival.

## Data Availability

No new data were created or analyzed in this study.
